# Cost‐Effectiveness Analysis of Fecal Immunochemical Test‐ and Colonoscopy‐based Colorectal Cancer Screening across Varying Uptake Rates

**DOI:** 10.1002/deo2.70236

**Published:** 2025-11-02

**Authors:** Masau Sekiguchi, Ataru Igarashi, Minoru esaki, Yutaka saito, Nozomu Kobayashi, Takahisa Matsuda

**Affiliations:** ^1^ Division of Screening Technology National Cancer Center Institute for Cancer Control Tokyo Japan; ^2^ Department of Endoscopy Gastrointestinal Endoscopy Division National Cancer Center Hospital Tokyo Japan; ^3^ Cancer Screening Center National Cancer Center Hospital Tokyo Japan; ^4^ Unit of Public Health and Preventive Medicine, School of Medicine Yokohama City University Kanagawa Japan; ^5^ Department of Health Economics and Outcomes Research, Graduate School of Pharmaceutical Sciences The University of Tokyo Tokyo Japan; ^6^ Department of Hepatobiliary and Pancreatic Surgery National Cancer Center Hospital Tokyo Japan; ^7^ Division of Gastroenterology and Hepatology Toho University Omori Medical Center Tokyo Japan

**Keywords:** colonoscopy, colorectal cancer screening, cost‐effectiveness, fecal immunochemical test, screening adherence

## Abstract

**Objectives:**

To reduce the burden of colorectal cancer (CRC), identifying cost‐effective screening strategies with particular attention to screening adherence is essential. We aimed to assess the cost‐effectiveness of screening strategies using fecal immunochemical test (FIT) and/or total colonoscopy (TCS) through simulation model analyses using Japanese data, considering various uptake rates.

**Methods:**

A state‐transition Markov model was used to evaluate the cost‐effectiveness of FIT‐based, TCS‐based, and combined FIT‐ and TCS‐based screening strategies. Variable uptake rates for FIT (40%–80%), screening TCS (10%–80%), and TCS following a positive FIT (70%–90%) were assessed. Analyses were performed from the healthcare payer's perspective, evaluating direct medical costs and quality‐adjusted life‐years (QALYs). Scenario analyses assuming substantially higher treatment costs for advanced CRC and probabilistic sensitivity analyses (PSA) for key parameters were also conducted.

**Results:**

Base‐case analyses demonstrated that higher screening uptake improved cost‐effectiveness across all strategies, with incremental cost‐effectiveness ratios (ICERs) below the 5,000,000 JPY threshold. In FIT‐based screening, increased uptake of TCS following a positive FIT improved QALYs and reduced costs. TCS‐based screening became more cost‐effective than FIT‐based screening when screening uptake was high (≥60%), with ICERs below the threshold; at lower uptake levels, FIT‐based screening remained superior. The combined strategy also required high uptake of screening TCS to surpass FIT‐based screening in cost‐effectiveness. Scenario analyses and PSA confirmed similar trends.

**Discussion:**

Our analyses highlight the critical impact of uptake rates on the cost‐effectiveness of CRC screening. Under currently realistic uptake conditions, FIT‐based screening remains the most cost‐effective strategy.

## 1 Introduction

Colorectal cancer (CRC) has imposed a significant global health burden due to its high incidence and mortality rates [1]. To reduce this burden, CRC screening programs are widely implemented [2]. Among these, the programs using the fecal immunochemical test (FIT) are the most widely adopted in the world, including in Japan [2]. Despite substantial evidence supporting the effectiveness of FIT‐based screening, the test has limited sensitivity for detecting advanced colorectal neoplasia in the proximal colon and serrated lesions [3, 4, 5, 6]. To address this limitation, FIT screening is typically conducted annually or biennially; however, the occurrence of interval cancers remains a notable concern [7, 8].

Screening programs that employ screening total colonoscopy (TCS) as an initial approach have the potential to overcome this limitation, as TCS allows for direct visualization of the whole intestinal mucosa and enables simultaneous polypectomy. Increasing evidence has demonstrated the effectiveness of TCS‐based screening programs [9, 10]. However, the invasive nature of TCS poses a risk of low adherence to TCS‐based screening programs [11, 12]. As suggested by the 10‐year results of the NORDICC study, low adherence can diminish the effectiveness of TCS‐based screening programs [13]. We further assume that adherence to CRC screening programs significantly affects their cost‐effectiveness, which is a critical factor for consideration.

It is important to evaluate the optimal CRC screening program by considering effectiveness and cost‐effectiveness, with particular attention to screening adherence. While several novel screening modalities are under development, the current lack of robust evidence supporting screening approaches using them underscores the need to optimize the use of established modalities, such as FIT and TCS, for CRC screening.

In this study, to explore an optimal CRC screening approach, we aimed to assess the cost‐effectiveness of screening strategies using FIT and/or TCS through simulation model analyses using Japanese data, with careful consideration of various uptake rates for screening and secondary examinations. Specifically, we evaluated FIT‐ and screening TCS‐based screening programs, as well as a hybrid strategy that combined FIT‐ and TCS‐based approaches.

## 2 Methods

### 2.1 Study Design

We performed a simulation model analysis using a Monte Carlo simulation to examine the cost‐effectiveness of CRC screening strategies using FIT and TCS. A previously developed and validated state‐transition Markov model of CRC progression was employed for this study [14, 15, 16, 17]. The model simulated CRC development based on the adenoma–carcinoma sequence, representing transitions from normal epithelium to low‐risk polyps (1–4 mm and 5–9 mm), high‐risk polyps, and CRC (Figure S1). CRC was defined as a malignant epithelial tumor of the large bowel that invaded beyond the muscularis mucosa [18]. Low‐risk polyps corresponded to non‐advanced adenomas, and high‐risk polyps to advanced adenomas, defined as adenomas with a diameter of ≥10 mm, high‐grade dysplasia, or a substantial villous component [19].

The initial simulated population comprised average‐risk Japanese individuals aged 40–74 years. Age and sex distributions were determined based on data from a screened Japanese population, as reported by the Japanese Society of Gastrointestinal Cancer Screening [20]. All individuals were assumed to be invited to CRC screening and to undergo screening according to a predefined uptake rate set in the model. The model used a one‐year cycle during which individuals moved to a different Markov status or stayed at the same status, and the analysis was conducted over each individual's lifetime.

The parameters used in this study, including transition probabilities and test characteristics, were primarily based on Japanese data, as detailed in our recent studies (Table S1) [14, 15, 16, 17]. Costs were determined using the Japanese medical care fee schedule and partly expert consensus [14, 15, 16, 17]. All costs are presented in both Japanese yen (JPY) and U.S. dollars (USD), based on the exchange rate as of May 9, 2025 (1 USD = 145.52 JPY).

### 2.2 Assessed CRC Screening Strategies and Uptake Rates

**FIGURE 1:**
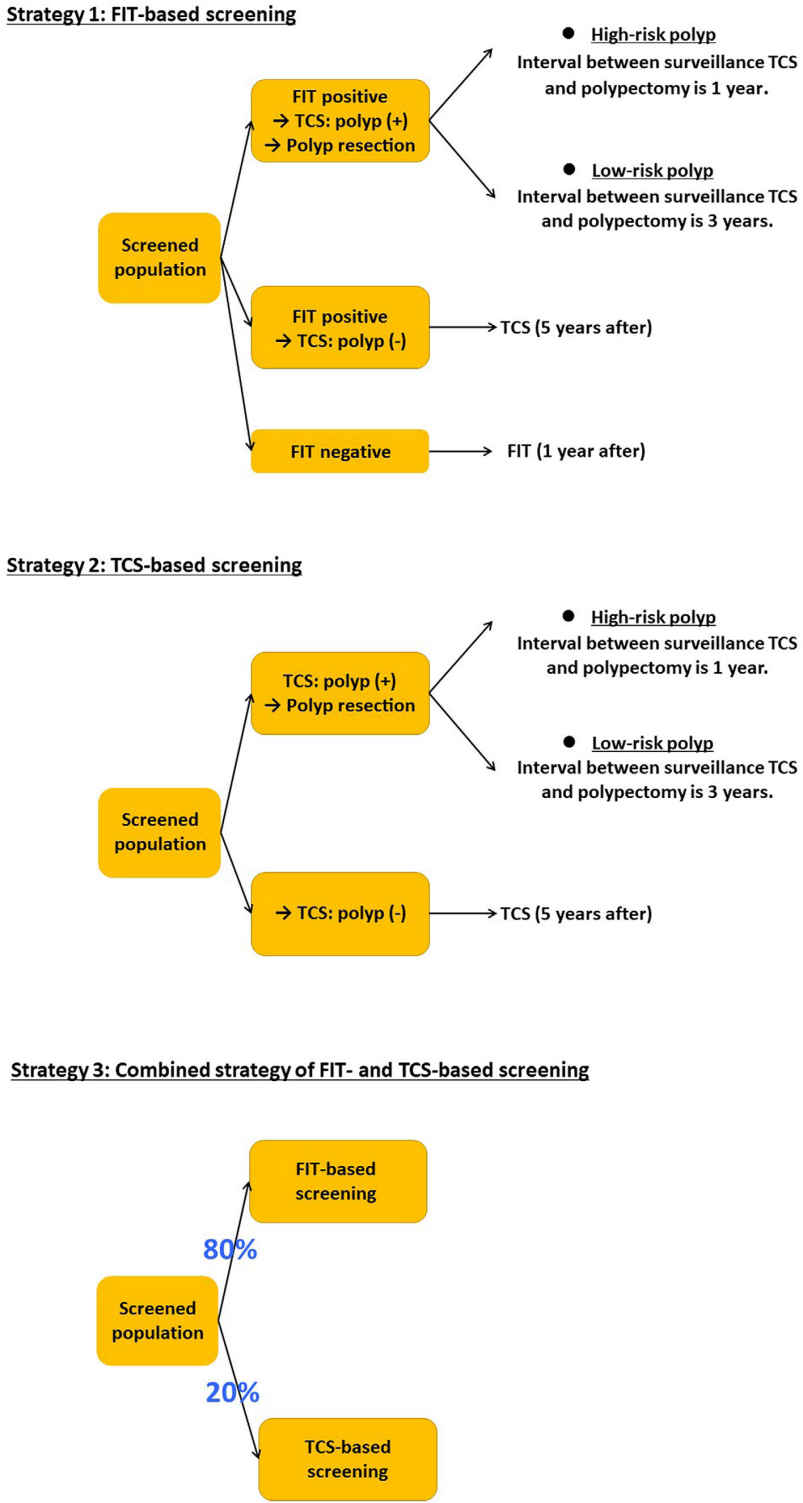
Colorectal cancer screening strategies assessed in this study. *FIT*, fecal immunochemical test; *TCS*, total colonoscopy.

Three CRC screening strategies, a FIT‐based strategy (Strategy 1), a TCS‐based strategy (Strategy 2), and a combined FIT‐ and TCS‐based strategy (Strategy 3), each with varying uptake rates, were assessed (Figure 1). In Strategies 1 and 2, FIT and TCS were offered, respectively, as the primary screening modality. In Strategy 1, individuals with a positive FIT result were referred for TCS. In Strategy 2, TCS was offered as a primary screening tool. In Strategy 3, FIT‐based and TCS‐based screening were offered to 80% and 20% of the population, respectively. This distribution was hypothetically determined, taking into account the reported preference for noninvasive tests [11, 12]. In all strategies, detected polyps were removed during TCS. The follow‐up and surveillance schedule adhered to the current Japanese guidelines [21].

In Strategy 1, FIT screening uptake rates ranging from 40% to 80% were examined to reflect the current uptake rate in Japanese population‐based screening and a higher, ideal scenario [22]. Uptake rates of 70% and 90% for TCS following a positive FIT were assessed, similarly reflecting current and ideal uptake conditions [22]. In Strategy 2, considering the reported tendency toward low adherence to primary TCS screening, uptake rates ranging from very low (10%) to high (80%) were evaluated [11, 12]. In Strategy 3, FIT‐based screening assumed a FIT uptake rate of 40% and a TCS uptake rate of 70% following a positive FIT. For TCS‐based screening within this strategy, primary TCS uptake rates ranging from 20% to 80% were assessed. In all strategies, the uptake rate for surveillance TCS was uniformly set at 70% to avoid its influence on outcomes when comparing the cost‐effectiveness of different CRC screening strategies.

### 2.3 Cost‐Effectiveness Analysis

We performed cost‐effectiveness analyses from the healthcare payer's perspective by performing a simulation model analysis using TreeAge Pro (TreeAge Software Inc., Williamstown, MA, USA). Only direct medical costs, including those for examinations and for the treatment of polyps and CRC, were considered. Effectiveness was measured in quality‐adjusted life‐years (QALYs), and outcomes such as CRC incidence and mortality were also evaluated.

We examined the cost‐effectiveness of each CRC screening strategy under varying uptake rates. In addition, we compared the cost‐effectiveness across different screening strategies. When a strategy was both less costly and more effective than another, it was considered clearly superior in cost‐effectiveness by simple dominance. If a strategy was more effective but more costly, its cost‐effectiveness was assessed by calculating the incremental cost‐effectiveness ratio (ICER), defined as the additional cost per QALY gained. The willingness‐to‐pay threshold was conservatively set at 5,000,000 JPY (34,359.5 USD) per QALY in this study [14, 15, 16, 17]. Both costs and effectiveness were discounted at an annual rate of 2%.

In addition to the base‐case analyses, we conducted a scenario analysis regarding the treatment cost of Dukes’ D CRC. Specifically, we explored the scenario in which this cost was set at ten times the base‐case value, to reflect recent substantial increases in chemotherapy expenses [23].

To compare the cost‐effectiveness of FIT‐based versus TCS‐based screening, we also conducted a probabilistic sensitivity analysis (PSA) for key parameters, including transition probabilities, costs, and test characteristics. The PSA was performed under two uptake scenarios: i) a realistic scenario based on current trends in Japan [20], assuming a 40% uptake rate for FIT, 70% for TCS following a positive FIT, and 20% for primary screening TCS; and ii) an optimistic scenario assuming improved screening participation, with 60% uptake rates for both FIT and primary screening TCS, and 70% for TCS following a positive FIT. This analysis involved 2000 Monte Carlo simulations. We applied beta and gamma distributions with a range of ± 25% for the parameters for which raw data of the denominators and numerators were available, such as test sensitivities, probability of colonoscopy perforation, and probability of development of new adenomas following endoscopic resection, and for the remaining variables, respectively, in line with our recent studies [14, 15, 16, 17].

## 3 RESULTS

### 3.1 Base‐Case Analysis for FIT‐based Screening Under Variable Uptake Rates

The cost‐effectiveness results for FIT‐based screening under variable uptake rates (40%–80% for FIT; 70% and 90% for TCS following a positive FIT) are summarized in Table 1. Comparisons were made against a reference scenario of FIT‐based screening with a 40% FIT uptake rate and a 70% TCS uptake rate following a positive FIT. Higher FIT screening uptake demonstrated increased costs but also greater effectiveness. Compared to a 40% uptake rate, higher uptake rates yielded ICERs well below the threshold (5,000,000 JPY; 34,359.5 USD), indicating superior cost‐effectiveness. When the TCS uptake rate following a positive FIT increased from 70% to 90%, both cost savings and improved effectiveness were observed, indicating superior cost‐effectiveness by simple dominance.

**TABLE 1:** Base‐case analysis for fecal immunochemical test‐based screening under variable uptake rates.

Uptake rate for FIT	40%	50%	60%	80%	40%	50%	60%	80%
Uptake rate for TCS following FIT+	70%				90%			
Cost (per person)	120,219 JPY (826.1 USD)	120,455 JPY (827.8 USD)	122,164 JPY (839.5 USD)	124,355 JPY (854.6 USD)	107,507 JPY (738.8 USD)	107,486 JPY (738.6 USD)	109,801 JPY (754.5 USD)	113,241 JPY (778.2 USD)
CRC cases (per 10^5^ persons)	2,644	2,466	2,353	2,183	2,051	1,835	1,691	1,508
CRC death (per 10^5^ persons)	665	608	582	548	428	373	355	324
QALYs (per person)	20.3912	20.4286	20.4358	20.4619	20.4379	20.4724	20.4844	20.502
ICER (per QALY) (vs. the scenario with uptake rates of 40% for FIT and 70% for TCS following FIT+	−	6,310 JPY (43.3 USD)	43,609 JPY (299.7 USD)	58,500 JPY (402.0 USD)	Dominant	Dominant	Dominant	Dominant

Abbreviations: *FIT*, fecal immunochemical test; *ICER*, incremental cost‐effectiveness ratio; QALY, quality‐adjusted life years; *TCS*, total colonoscopy.

### 3.2 Base‐Case Analysis for TCS‐based Screening Under Variable Uptake Rates

Table 2 presents the results for TCS‐based screening under variable screening uptake rates (10%–80%). Comparisons with FIT‐based screening at equivalent uptake rates and with a 70% TCS uptake rate following a positive FIT are also included. As screening uptake increased, TCS‐based screening demonstrated both cost reductions and improved effectiveness. When comparing with FIT‐based screening, TCS‐based screening was more cost‐effective at uptake rates of 60% or higher, with ICERs well below the threshold. At lower uptake rates, FIT‐based screening was more cost‐effective than TCS‐based screening by simple dominance.

**TABLE 2:** Base‐case analysis for colonoscopy‐based screening under variable uptake rates.

Uptake rate for primary screening TCS	10%	20%	40%	50%	60%	80%
Cost (per person)	181,575 JPY (1247.8 USD)	164,884 JPY (1133.1 USD)	144,625 JPY (993.8 USD)	137,545 JPY (945.2 USD)	133,121 JPY (914.8 USD)	130,661 JPY (897.9 USD)
CRC cases (per 10^5^ persons)	5,266	4,112	2,576	2,144	1,828	1,236
CRC death (per 10^5^ persons)	1,808	1,375	764	625	513	337
QALYs (per person)	20.1965	20.2856	20.3895	20.4243	20.4649	20.4995
ICER (per QALY) (vs FIT screening under the same screening uptake rate and the uptake of 70% for TCS following FIT+)	Dominated	Dominated	Dominated	Dominated	376,529 JPY (2587.5 USD)	167,713 JPY (1152.5 USD)

Abbreviations: *FIT*, fecal immunochemical test; *ICER*, incremental cost‐effectiveness ratio; QALY, quality‐adjusted life years; *TCS*, total colonoscopy.

### 3.3 Base‐Case Analysis for Combined FIT‐ and TCS‐based Screening Under Variable Uptake Rates

Table 3 summarizes the results for combined FIT‐ and TCS‐based screening under varying uptake scenarios. Comparisons were made against a reference scenario of FIT‐based screening with a 40% FIT uptake rate and a 70% TCS uptake rate following a positive FIT. The combined strategy showed superior cost‐effectiveness compared to FIT‐based screening when the TCS‐based screening group achieved an uptake rate of 60% or higher. At lower uptake rates in the TCS‐based group, the FIT‐based screening strategy remained more cost‐effective than the combined screening strategy.

**TABLE 3:** Base‐case analysis for combined fecal immunochemical test‐ and colonoscopy‐based screening under variable uptake rates.

Uptake rates for FIT and TCS following a positive FIT in the FIT‐based screening group	FIT: 40%, TCS for FIT+: 70%			
Uptake rate for screening TCS in the TCS‐based screening group	20%	40%	60%	80%
Cost (per person)	129,152 JPY (887.5 USD)	125,100 JPY (859.7 USD)	122,799 JPY (843.9 USD)	116,335 JPY (799.4 USD)
CRC cases (per 10^5^ persons)	2,949	2,654	2,498	2,104
CRC death (per 10^5^ persons)	799	698	642	489
QALYs (per person)	20.3701	20.3909	20.4059	20.4129
ICER (per QALY) (vs FIT screening under uptake rates of 40% for FIT and 70% for TCS following FIT+)	Dominated	Dominated	175,869 JPY (1208.6 USD)	Dominant

Abbreviations: *FIT*, fecal immunochemical test; *ICER*, incremental cost‐effectiveness ratio; QALY, quality‐adjusted life years; *TCS*, total colonoscopy.

### 3.4 Scenario Analyses With Substantially Increased Costs for Advanced CRC Treatment

The cost‐effectiveness results for screening strategies 1–3, assuming a tenfold increase in the treatment cost of Dukes' D CRC, are presented in Tables S2–S4, respectively. The results demonstrated trends consistent with those observed in the base‐case analyses, with comparable patterns in cost‐effectiveness across varying uptake rates and among the different screening strategies.

### 3.5 PSA Comparing the Cost‐effectiveness of FIT‐based and TCS‐based Screening

**FIGURE 2:**
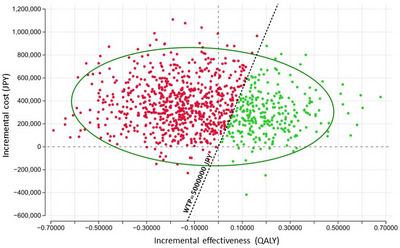
Probabilistic sensitivity analysis comparing the cost‐effectiveness of fecal immunochemical test‐based and colonoscopy‐based screening strategies under a realistic uptake scenario. Scatter plot showing the results of 2000 Monte Carlo simulations, assuming a 40% fecal immunochemical test (FIT) uptake rate, 70% total colonoscopy (TCS) uptake after a positive FIT, and 20% uptake for primary TCS screening. Green plots (27.6%) indicate the simulations in which TCS‐based screening was more cost‐effective than FIT‐based screening under the willingness‐to‐pay value of 5,000,000 JPY (34,359.5 USD). *QALY*, quality‐adjusted life‐year; *WTP*, willingness‐to‐pay.

**FIGURE 3:**
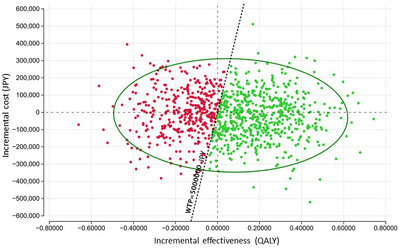
Probabilistic sensitivity analysis comparing the cost‐effectiveness of fecal immunochemical test‐based and colonoscopy‐based screening strategies under an optimistic uptake scenario. Scatter plot showing the results of 2000 Monte Carlo simulations, assuming a 60% fecal immunochemical test (FIT) uptake rate, 70% total colonoscopy (TCS) uptake after a positive FIT, and 60% uptake for primary TCS screening. Green plots (61.6%) indicate the simulations in which TCS‐based screening was more cost‐effective than FIT‐based screening under the willingness‐to‐pay value of 5,000,000 JPY (34,359.5 USD). *QALY*, quality‐adjusted life‐year; *WTP*, willingness‐to‐pay.

The results of the PSA comparing the cost‐effectiveness of FIT‐based and TCS‐based screening under the two uptake scenarios are illustrated as scatter plots of 2,000 Monte Carlo simulations on the cost‐effectiveness plane (Figures 2 and 3). Under a willingness‐to‐pay threshold of 5,000,000 JPY (34,359.5 USD) per QALY, the probability of TCS‐based screening being more cost‐effective than FIT‐based screening was 27.6% in the realistic uptake scenario and 61.6% in the optimistic scenario reflecting improved screening participation.

## 4 Discussion

In this study, we examined the cost‐effectiveness of CRC screening strategies using FIT and/or TCS under various uptake rates. This study has several notable strengths. We employed an established simulation model with parameters previously developed and validated in our recent studies [14, 15, 16, 17]. A broad spectrum of uptake scenarios was analyzed for both primary screening and secondary examinations, ranging from realistic to optimal participation rates. We also conducted scenario analyses assuming substantially increased treatment costs for advanced CRC, considering the recent increase in chemotherapy costs [23]. Furthermore, we performed probabilistic sensitivity analyses to account for uncertainties in model parameters. These strengths enabled a comprehensive evaluation of how uptake rates affect cost‐effectiveness and provided valuable insights into the comparative value of CRC screening strategies under varying levels of participation.

Our results clearly highlight the importance of screening uptake rates for the effectiveness and cost‐effectiveness of CRC screening. In all screening strategies (FIT‐based, TCS‐based, and combined), higher screening uptake was associated with improved effectiveness and cost‐effectiveness. Notably, the remarkable impact of the uptake rate for TCS following a positive FIT on the cost‐effectiveness of FIT‐based screening was also emphasized. When comparing different screening strategies, our findings suggest that TCS‐based screening can be more effective and cost‐effective than FIT‐based screening if a sufficiently high screening uptake rate (≥60%) is achievable. However, under currently realistic uptake conditions, FIT‐based screening remains the more cost‐effective option. Given the substantial heterogeneity in previous literature regarding the cost‐effectiveness of CRC screening strategies, our clear and straightforward findings, achieved through careful attention to screening adherence, provide valuable and actionable insights [24, 25].

Considering the tendency of low uptake rate for primary screening TCS, we suggest that, in pursuit of an optimal and realistic CRC screening strategy in terms of cost‐effectiveness, efforts should primarily focus on enhancing the FIT‐based screening program [11, 12, 13]. Improving the uptake rate for TCS following a positive FIT in the program is especially crucial. Our suggestion aligns with recent clinical trial results showing suboptimal effectiveness of TCS‐based screening when uptake rates are inadequate, and demonstrating noninferiority of FIT‐based screening to TCS‐based screening when uptake is higher [13, 26]. Considering that real‐world uptake for primary TCS screening may be even lower than in clinical trial settings, our support for a FIT‐based approach remains valid. Nevertheless, our findings do not exclude the potential value of implementing TCS‐based screening in population‐based programs. If introduced in a safe, structured manner that ensures high participation, TCS‐based screening could play a beneficial role. In this context, a combined approach using both FIT‐ and TCS‐based strategies (such as Strategy 3 with a high uptake rate in the TCS‐based screening group) or a risk‐stratified model incorporating both strategies may represent a helpful direction [5, 27, 28].

This study has several limitations. Some degree of uncertainty in model parameters was unavoidable. However, the scenario analyses and PSA we performed support the robustness of our findings. The generalizability of the results may be limited, as the analyses were primarily based on Japanese data. Nevertheless, the findings align with recent clinical trial results from other countries, suggesting reasonable generalizability [13, 26]. Additionally, our model did not incorporate the serrated pathway in colorectal carcinogenesis.

In conclusion, our analyses highlight the critical impact of uptake rates on the cost‐effectiveness of CRC screening. Under currently realistic screening uptake conditions, FIT‐based screening remains the most cost‐effective option. Therefore, prioritizing the enhancement of FIT‐based programs, particularly through increasing the uptake rate for TCS following a positive FIT, appears to be a reasonable approach.

## Author Contributions

**Masau Sekiguchi**: conceptualization; data curation; funding acquisition; investigation; methodology; project administration; visualization; writing – original draft; writing – review and editing. **Ataru Igarashi**: conceptualization; data curation; formal analysis; investigation; methodology; software; visualization; writing ‐ review and editing. **Minoru Esaki**: conceptualization; funding acquisition; investigation; writing – review and editing. **Yutaka Saito**: conceptualization; investigation; writing – review and editing. **Nozomu Kobayashi**: funding acquisition; investigation; writing – review and editing. **Takahisa Matsuda**: conceptualization; investigation; methodology; writing – review and editing.

## Conflicts of Interest

The authors declare no conflicts of interest.

## Funding

This study was supported by the National Cancer Center Research and Development Fund (2025‐A‐21 and 2024‐A‐16).

## Ethics Statement

Approval of the research protocol by an Institutional Reviewer Board: N/A

## Consent

N/A.

## Clinical Trial Registration

N/A.

## Supporting information




**FIGURE S1** Colorectal cancer model used in this study. *CRC*, colorectal cancer.

**TABLE S1** Model parameters used in the model analysis. (Table modified from Table 1 in Ref. 14, Table 1 in Ref. 15, and Table S1 in Ref. 16).
**TABLE S2** Scenario analysis for fecal immunochemical test‐based screening under variable uptake rates. *FIT*, fecal immunochemical test; *ICER*, incremental cost‐effectiveness ratio; QALY, quality‐adjusted life years; *TCS*, total colonoscopy.
**TABLE S3** Scenario analysis for colonoscopy‐based screening under variable uptake rates. *FIT*, fecal immunochemical test; *ICER*, incremental cost‐effectiveness ratio; QALY, quality‐adjusted life years; *TCS*, total colonoscopy.
**TABLE S4** Scenario analysis for combined fecal immunochemical test‐ and colonoscopy‐based screening under variable uptake rates. *FIT*, fecal immunochemical test; *ICER*, incremental cost‐effectiveness ratio; QALY, quality‐adjusted life years; *TCS*, total colonoscopy.
