# Supplementary figures and images for: Cost‐Effectiveness Analysis of Fecal Immunochemical Test‐ and Colonoscopy‐based Colorectal Cancer Screening across Varying Uptake Rates
Source: DEN Open. 2025 Nov 2;6(1):e70236. doi: 10.1002/deo2.70236 (PMC12580226; doi:10.1002/deo2.70236)

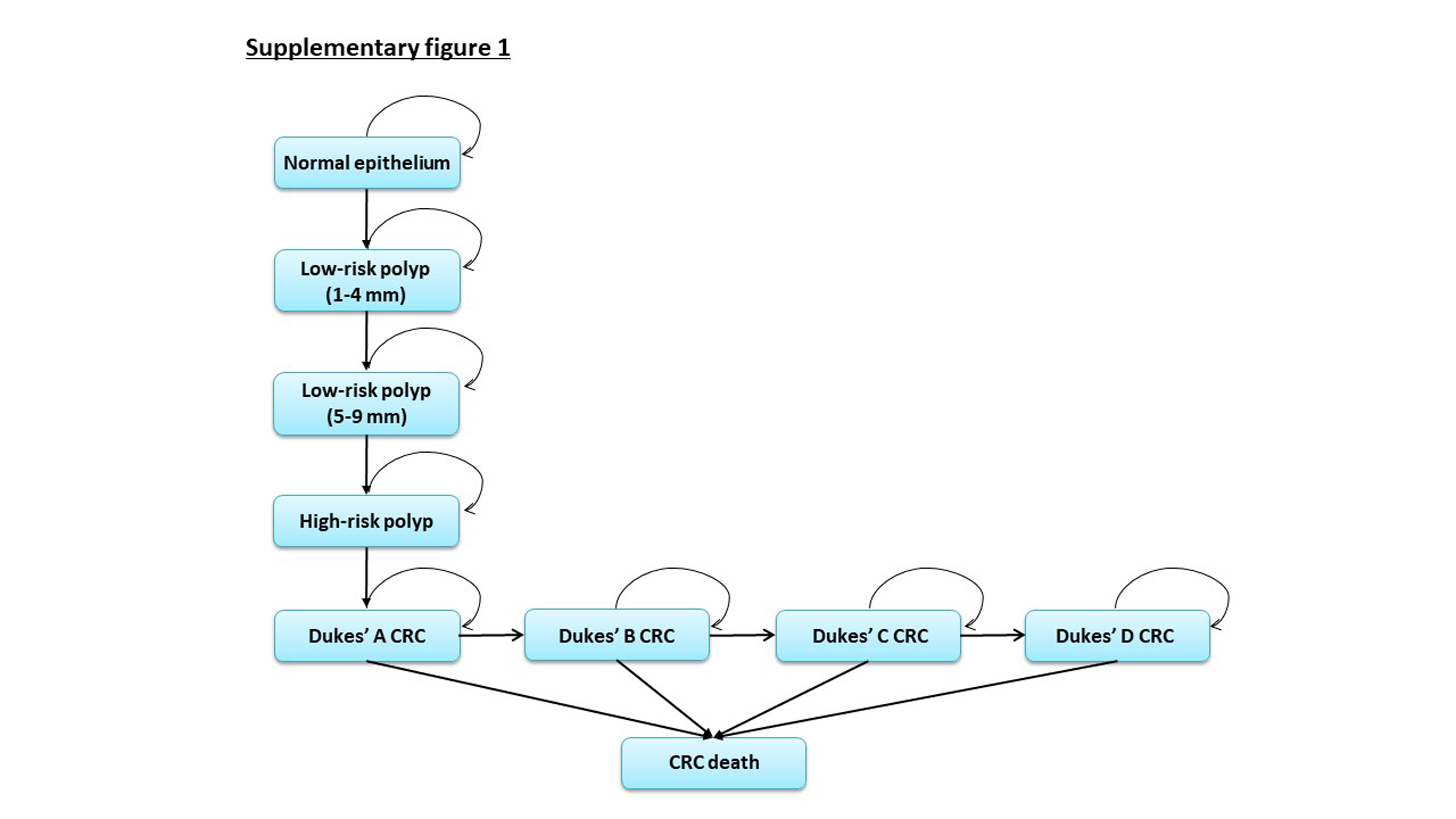

Supplement: Supplementary file 1 — FIGURE S1 Colorectal cancer model used in this study. CRC, colorectal cancer. [file DEO2-6-e70236-s001.tif]
