# Supplementary material for: Cost‐Effectiveness Analysis of Fecal Immunochemical Test‐ and Colonoscopy‐based Colorectal Cancer Screening across Varying Uptake Rates
Source: DEN Open. 2025 Nov 2;6(1):e70236. doi: 10.1002/deo2.70236 (PMC12580226; doi:10.1002/deo2.70236)
Supplement: Supplementary file 2 — TABLE S1 Model parameters used in the model analysis. (Table modified from Table 1 in Ref. 14, Table 1 in Ref. 15, and Table S1 in Ref. 16). TABLE S2 Scenario analysis for fecal immunochemical test‐based screening under variable uptake rates. FIT, fecal immunochemical test; ICER, incremental cost‐effectiveness ratio; QALY, quality‐adjusted life years; TCS, total colonoscopy. TABLE S3 Scenario analysis for colonoscopy‐based screening under variable uptake rates. FIT, fecal immunochemical test; ICER, incremental cost‐effectiveness ratio; QALY, quality‐adjusted life years; TCS, total colonoscopy. TABLE S4 Scenario analysis for combined fecal immunochemical test‐ and colonoscopy‐based screening under variable uptake rates. FIT, fecal immunochemical test; ICER, incremental cost‐effectiveness ratio; QALY, quality‐adjusted life years; TCS, total colonoscopy. [file DEO2-6-e70236-s002.docx]

**Supplementary Table 1.** Model parameters used in the model analysis (table modified from Table 1 in Ref. 14, Table 1 in Ref. 15, and supplementary Table 1 in Ref 16)

| **Model parameters** | **Baseline value** |
| --- | --- |
| **Transition probabilities (per year)** |  |
| **Probability of progression in colorectal cancer development** |  |
| From normal epithelium to low-risk polyp (1–4mm) | 3.4–6.6%  (varies with age) |
| From low-risk polyp (1–4 mm) to low-risk polyp (5–9 mm) | 1.4–5.6%  (varies with age) |
| From low-risk polyp (5–9 mm) to high-risk polyp | 1.3–5.6%  (varies with age) |
| From high-risk polyp to Dukes’ A colorectal cancer | 3.4% |
| From Dukes’ A colorectal cancer to Dukes’ B | 58.3% |
| From Dukes’ B colorectal cancer to Dukes’ C | 65.6% |
| From Dukes’ C colorectal cancer to Dukes’ D | 86.5% |
| **Probability of death from colorectal cancer** |  |
| Dukes’ A | 1.7% |
| Dukes’ B | 3.2% |
| Dukes’ C | 7.2% |
| Dukes’ D | 28.4% |
| **Probability of symptomatic presentation of colorectal cancer** |  |
| Dukes’ A | 6.5% |
| Dukes’ B | 26.0% |
| Dukes’ C | 46.0% |
| Dukes’ D | 92.0% |
| **Probability of developing new polyp following endoscopic resection** |  |
| Developing low-risk polyp (1–4mm) following endoscopic resection | 10.0% |
| Developing low-risk polyp (5–9mm) following endoscopic resection | 5.3% |
| Developing high-risk polyp following endoscopic resection | 0.7% |
| **Probability of recurrence following treatment of colorectal cancer** |  |
| Dukes’ A | 0.8% |
| Dukes’ B | 2.8% |
| Dukes’ C | 7.1% |
|  |  |
| **Cost** |  |
| Fecal immunochemical test | 1,600 JPY/ 11.0 USD |
| Screening colonoscopy | 15,500 JPY/ 106.5 USD |
| Endoscopic resection of low-risk polyp | 50,000 JPY/ 343.6 USD |
| Endoscopic resection of high-risk polyp | 157,114 JPY/ 1,079.7 USD |
| **Annual cost for colorectal cancer treatment** |  |
| Dukes’ A (1y) | 1,319,816 JPY/ 9,069.7 USD |
| Dukes’ A (2–5y) | 35,570 JPY/ 244.4 USD |
| Dukes’ B (1y) | 1,399,034 JPY/ 9,614.0 USD |
| Dukes’ B (2–5y) | 35,570 JPY/ 244.4 USD |
| Dukes’ C (1y) | 2,340,416 JPY/ 16,083.1 USD |
| Dukes’ C (2–5y) | 44,972 JPY/ 309.0 USD |
| Dukes’ D (1y) | 2,687,125 JPY/ 18,465.7 USD |
| Dukes’ D (2–5y) | 2,544,972 JPY/ 17,488.8 USD |
|  |  |
| **Test characteristics** |  |
| **Fecal immunochemical test** |  |
| Sensitivity for low-risk polyp (1–4 mm) | 6.3% |
| Sensitivity for low-risk polyp (5–9 mm) | 7.9% |
| Sensitivity for high-risk polyp | 26.5% |
| Sensitivity for Dukes’ A colorectal cancer | 52.8% |
| Sensitivity for Dukes’ B colorectal cancer | 70.0% |
| Sensitivity for Dukes’ C and D colorectal cancer | 78.3% |
| Specificity for colorectal polyp and colorectal cancer | 94.6% |
| **Total colonoscopy** |  |
| Sensitivity for low-risk polyp (1–4 mm) | 74.1% |
| Sensitivity for low-risk polyp (5–9 mm) | 86.5% |
| Sensitivity for high-risk polyp | 97.6% |
| Sensitivity for colorectal cancer (Dukes’ A–D) | 99.9% |
| Specificity for colorectal polyp and colorectal cancer | 100.0% |
| Probability of perforation from colonoscopy without endoscopic resection | 0.01% |
| Probability of perforation from colonoscopy with endoscopic resection | 0.06% |
| Probability of death following perforation | 6.7% |
| Probability of bleeding from colonoscopy with endoscopic resection | 0.5% |

**Supplementary Table 2**. Scenario analysis for fecal immunochemical test-based screening under variable uptake rates

| Uptake rate for FIT | 40% | 50% | 60% | 80% | 40% | 50% | 60% | 80% |
| --- | --- | --- | --- | --- | --- | --- | --- | --- |
| Uptake rate for TCS following FIT+ | 70% | | | | 90% | | | |
| Cost (per person) | 370,871 JPY  (2548.6 USD) | 350,968 JPY  (2411.8 USD) | 344,040 JPY  (2364.2 USD) | 332,887 JPY  (2287.6 USD) | 252,303 JPY  (1733.8 USD) | 228,268 JPY  (1568.6 USD) | 225,956 JPY  (1552.7 USD) | 218,619 JPY  (1502.3 USD) |
| CRC cases (per 10^5^ persons) | 2,644 | 2,466 | 2,353 | 2,183 | 2,051 | 1,835 | 1,691 | 1,508 |
| CRC death (per 10^5^ persons) | 665 | 608 | 582 | 548 | 428 | 373 | 355 | 324 |
| QALYs (per person) | 20.3912 | 20.4286 | 20.4358 | 20.4619 | 20.4379 | 20.4724 | 20.4844 | 20.502 |
| ICER (per QALY) (vs the scenario with uptake rates of 40% for FIT and 70% for TCS following FIT+ | － | Dominant | Dominant | Dominant | Dominant | Dominant | Dominant | Dominant |

*FIT*, fecal immunochemical test; *ICER*, incremental cost-effectiveness ratio; QALY, quality-adjusted life years; *TCS*, total colonoscopy.

**Supplementary Table 3**. Scenario analysis for colonoscopy-based screening under variable uptake rates

| Uptake rate for primary screening TCS | 10% | 20% | 40% | 50% | 60% | 80% |
| --- | --- | --- | --- | --- | --- | --- |
| Cost (per person) | 943,067 JPY  (6480.7 USD) | 735,476 JPY  (5054.1 USD) | 468,236 JPY  (3217.7 USD) | 377,305 JPY  (2592.8 USD) | 318,406 JPY  (2188.1 USD) | 237,789 JPY  (1634.1 USD) |
| CRC cases (per 10^5^ persons) | 5,266 | 4,112 | 2,576 | 2,144 | 1,828 | 1,236 |
| CRC death (per 10^5^ persons) | 1,808 | 1,375 | 764 | 625 | 513 | 337 |
| QALYs (per person) | 20.1965 | 20.2856 | 20.3895 | 20.4243 | 20.4649 | 20.4995 |
| ICER (per QALY) (vs FIT screening under the same screening uptake rate and the uptake of 70% for TCS following FIT+) | Dominated | Dominated | Dominated | Dominated | Dominant | Dominant |

*FIT*, fecal immunochemical test; *ICER*, incremental cost-effectiveness ratio; QALY, quality-adjusted life years; *TCS*, total colonoscopy.

**Supplementary Table 4**. Scenario analysis for combined fecal immunochemical test- and colonoscopy-based screening under variable uptake rates

| Uptake rates for FIT and TCS following a positive FIT in the FIT-based screening group | FIT: 40%, TCS for FIT+: 70% | | | |
| --- | --- | --- | --- | --- |
| Uptake rate for screening TCS in the TCS-based screening group | 20% | 40% | 60% | 80% |
| Cost (per person) | 443,792 JPY  (3049.7 USD) | 411,887 JPY  (2830.4 USD) | 347,519 JPY  (2388.1 USD) | 262,711 JPY  (1805.3 USD) |
| CRC cases (per 10^5^ persons) | 2,949 | 2,654 | 2,498 | 2,104 |
| CRC death (per 10^5^ persons) | 799 | 698 | 642 | 489 |
| QALYs (per person) | 20.3701 | 20.3909 | 20.4059 | 20.4129 |
| ICER (per QALY) (vs FIT screening under uptake rates of 40% for FIT and 70% for TCS following FIT+) | Dominated | Dominated | Dominant | Dominant |

*FIT*, fecal immunochemical test; *ICER*, incremental cost-effectiveness ratio; QALY, quality-adjusted life years; *TCS*, total colonoscopy.
